# Redox Signaling and CBF-Responsive Pathway Are Involved in Salicylic Acid-Improved Photosynthesis and Growth under Chilling Stress in Watermelon

**DOI:** 10.3389/fpls.2016.01519

**Published:** 2016-10-10

**Authors:** Fei Cheng, Junyang Lu, Min Gao, Kai Shi, Qiusheng Kong, Yuan Huang, Zhilong Bie

**Affiliations:** ^1^Key Laboratory of Horticultural Plant Biology, Ministry of Education/College of Horticulture and Forestry Sciences, Huazhong Agricultural UniversityWuhan, China; ^2^Department of Horticulture, Zhejiang UniversityHangzhou, China

**Keywords:** antioxidant metabolism, ascorbate, CBF-responsive pathway, chilling stress, *Citrullus lanatus*, glutathione, photosynthesis, salicylic acid

## Abstract

Salicylic acid (SA) plays an important role in plant response to abiotic stresses. This study investigated the potential role of SA in alleviating the adverse effects of chilling stress on photosynthesis and growth in watermelon (*Citrullus lanatus*). Chilling stress induced the simultaneous accumulation of free and conjugated SA in watermelon plants, and the chilling-induced SA production was attributed to the phenylalanine ammonia-lyase pathway. Applying SA at moderate concentrations induced chilling tolerance, whereas inhibition of SA biosynthesis by L-α-aminooxy-β-phenylpropionic acid (AOPP) increased the photooxidation of PS II under chilling stress in watermelon, resulting in reduced photosynthesis and growth. Chilling induced a transient increase in the ratios of reduced to oxidized glutathione and reduced ascorbate to dehydroascorbate. Then, the expression of antioxidant genes was upregulated, and the activities of antioxidant enzymes were enhanced. Furthermore, SA-induced chilling tolerance was associated with cellular glutathione and ascorbate homeostasis, which served as redox signals to regulate antioxidant metabolism under chilling stress. AOPP treatment stimulated the chilling-induced expression of cold-responsive genes, particularly via C-repeat binding factors CBF3 and CBF4. These results confirm the synergistic role of SA signaling and the CBF-dependent responsive pathway during chilling stress in watermelon.

## Introduction

Salicylic acid is a phenolic compound involved in the regulation of plant growth and development. SA also serves as a critical signal to activate disease resistance in *Arabidopsis thaliana* and other plant species. This compound induces defense-related genes and stress resistance in biotic stressed plants (Kumar, 2014) and improves plant tolerance to various abiotic stresses, such as heavy metal stress (Zhang et al., 2015), salinity (Khan et al., 2014), osmotic stress (Alavi et al., 2014), drought (Nazar et al., 2015), and heat stress (Khan et al., 2013). SA is synthesized via two distinct pathways, namely, the isochorismate (IC) pathway and the PAL pathway. Both of these pathways originate from chorismic acid, which is the end product of the shikimate pathway. In the IC pathway, chorismic acid is converted into IC by ICS. The conversion of IC into SA may be catalyzed by isochorismate pyruvate lyase (IPL) from *Pseudomonas aeruginosa* and *Pseudomonas fluorescens* (Serino et al., 1995; Mercado-Blanco et al., 2001). However, a plant gene that encodes a protein with IPL activity has not been identified. In the PAL pathway, PAL enzyme deaminates phenylalanine to form *trans*-cinnamic acid, which is then converted into SA via two possible intermediates, namely, *ortho*-coumaric acid and BA (Chadha and Brown, 1974; Yalpani et al., 1993). The conversion of BA into SA has been proposed to occur via an inducible BA2H.

Cold response is a highly complex process that involves an array of physiological and biochemical modifications. Chloroplast plays an important role in sensing ambient temperature. Under low temperature, an imbalance between the capacity to harvest and dissipate light energy through metabolic activity causes excess PS II excitation pressure, leading to the generation of ROS and impairment of photosynthesis. A recent study has reported that SA improves the growth and photosynthesis of several crop plants through osmolyte synthesis modulation, antioxidant system activation, secondary metabolite production, and mineral nutrient status optimization (review; Khan et al., 2015). Ascorbate and glutathione, as vital components of the redox hub, are multifunctional metabolites that play significant roles in redox homeostasis and signaling; these metabolites are also essential in the development and defense reactions of plants under biotic or abiotic stresses (Foyer and Noctor, 2011; Anjum et al., 2016). Controlled levels of SA are required for redox homeostasis. In *Arabidopsis* mutants, constitutive accumulation of SA strongly increases the levels of hydrogen peroxide (H_2_O_2_) and reduced glutathione under low-light control conditions; by contrast, low SA levels decrease H_2_O_2_ content and increase glutathione oxidation under low-light conditions (Mateo et al., 2006). SA-improved abiotic stress tolerance is associated with ascorbate–glutathione metabolism (Wang and Li, 2006; Li et al., 2013). Furthermore, ROS and glutathione are involved in the SA signaling pathway, which can induce systemic acquired resistance (Mou et al., 2003; Foyer and Noctor, 2005; Sewelam et al., 2016). The abundance of leaf ascorbate influences the SA signaling pathway because numerous transcripts that encode SA-inducible proteins are constitutively expressed in the ascorbate-deficient *Arabidopsis* mutants *vtc1* and *vtc2* (Kiddle et al., 2003; Pavet et al., 2005; Brosche and Kangasjarvi, 2012). However, the potential roles of ascorbate and glutathione in SA-induced chilling tolerance remain unknown.

Cold acclimation is regulated by the CBF/DREB1-dependent cold signaling pathway, which is controlled by an MYC-type transcription factor ICE1 (inducer of *CBF* expression1; Chinnusamy et al., 2003, 2007). In *Arabidopsis*, CBF1/DREB1B, CBF2/DREB1C, and CBF3/DREB1A are involved in the regulation of *COR* gene expression and cold tolerance (Gilmour et al., 2000, 2004). Among the phytohormones, ABA, auxin, gibberellin (GA), ethylene, and SA act as positive or negative modulators of cold responses. Endogenous free SA and glucosyl SA accumulate during chilling in *Arabidopsis*, cucumber, and wheat (Scott et al., 2004; Kosova et al., 2012; Dong et al., 2014). SA treatment also enhances the cold tolerance of rice, maize, and cucumber (Kang and Saltveit, 2002; Dong et al., 2014). However, a consensus regarding the regulation of CBFs by SA in response to chilling remains to be established.

Watermelon of the Cucurbitaceae family is an important economic crop worldwide. As a chilling-sensitive crop, watermelon usually suffers from chilling stress (<15°C) when grown in the winter or early spring. Phytohormones are effective in mitigating chilling injury in crop plants. In particular, SA improves plant chilling tolerance in various species. However, the molecular mechanisms underlying SA-mediated chilling tolerance remain unclear to date. To examine the role of SA in chilling tolerance, we analyzed the SA accumulation, transcript levels, and activities of the enzymes involved in SA biosynthesis and examined the antioxidant metabolism in response to chilling stress in watermelon. Furthermore, we determined the effects of chilling and SA treatment on chlorophyll fluorescence, electrolyte leakage, gas exchange and growth, glutathione and ascorbate contents, and *CBF* and *COR* gene expression to explore the relationship between cold signaling and SA signaling in watermelon plants.

## Materials and Methods

### Plant Materials and Experimental Design

In a growth chamber, watermelon [*Citrullus lanatus* (Thunb.) Matsum. & Nakai var. *lanatus* 97103] seeds were germinated in a growth medium composed of a mixture of peat and perlite (2:1, v:v). When the cotyledonary leaves were fully expanded, the seedlings were watered daily with Hoagland’s nutrient solution. The growth conditions were described as follows: a 12 h photoperiod, temperature of 28/18°C (day/night), and photosynthetic photon flux density (PPFD) of 300 μmol m^-2^ s^-1^.

Three experiments were performed in this study. In the first experiment, plants at the four-leaf stage were placed in growth chambers (Conviron E15; Conviron, MB, Canada) at 28/18°C (day/night) or 10/5°C (day/night) with 300 μmol m^-2^ s^-1^ PPFD for 7 days. Leaf samples harvested from chilling and untreated watermelon plants at different time points were frozen immediately in liquid nitrogen and then stored at -80°C prior to SA measurements. Subsequently, gene expression, enzyme activity, and cellular redox status analyses were conducted. In the second experiment, watermelon plants at the four-leaf stage were pretreated with different concentrations of SA. After 24 h, the SA-treated plants were placed in growth chambers at 10/5°C with 300 μmol m^-2^ s^-1^ PPFD for 7 days and then immediately analyzed for *F*v/*F*m or electrolyte leakage. In the third experiment, half of the watermelon plants at the four-leaf stage were sprayed with 50 μM AOPP, an SA biosynthesis inhibitor used to inhibit PAL activity (Dong et al., 2014). The other half of the plants were sprayed with water. After 1 day, the AOPP-treated and half of the water-treated plants were exposed to 10/5°C and 300 μmol m^-2^ s^-1^ PPFD for 5 days. The remaining water-treated plants (1/4 of the total) were maintained in a growth chamber at 28/18°C with 300 μmol m^-2^ s^-1^ PPFD to serve as the control group. At 1 day after chilling treatment, half of the AOPP-treated plants were sprayed with SA, and another half were treated with water followed by chilling treatment for 4 days. Leaf samples were harvested at different time points for chlorophyll fluorescence and gas exchange measurements and cellular redox status and gene expression analyses.

### SA Measurements

Salicylic acid measurements were conducted using a rapid biosensor-based method as described by Defraia et al. (2008). Leaf tissues were ground in liquid nitrogen and then left at room temperature for 5 min. Acetate buffer (0.1 M, pH 5.6) was added at a ratio of 2.5 μL/mg tissue at room temperature before samples were mixed and centrifuged for 15 min at 16,000 ×*g*. Half (100 μL) of the supernatant was stored on ice for free SA measurement, and the other half was incubated at 37°C for 90 min with 4 U of β-glucosidase (3.2.1.21, Sigma-Aldrich, St. Louis, MO, USA) for conjugated SA measurement.

An overnight biosensor culture of *Acinetobacter* sp. ADPWH_*lux* was diluted in 37°C LB (1:20) and grown for ∼3 h at 200 rpm to an OD600 of 0.4. Up to 20 μL of crude extract that was stored at room temperature (20–22°C) was added to 60 μL of LB and 50 μL of biosensor culture in a black 96-well cell culture plate. The plate was incubated at 37°C for 1 h without shaking before luminescence was read by on an Infinite M200 Pro Multi-Detection Microplate Reader (Tecan, Männedorf, Zürich, Switzerland).

### Determination of PAL and BA2H Activities

For the PAL and BA2H activities, leaf tissues (0.3 g) were ground in liquid nitrogen and then added with 1.5 mL of ice-cold buffer containing 50 mM Tris-HCl (pH 8.5), 5 mM EDTA, 15 mM β-mercaptoethanol, 1 mM 4-(2-Aminoethyl) benzenesulfonyl fluoride hydrochloride (AEBSF), and 0.15% (w/v) polyvinylpyrrolidone (PVP). Homogenates were centrifuged at 12,000 ×*g* for 20 min at 4°C, and the resulting supernatants were used to determine the enzyme activity. PAL activity was measured on the basis of the formation of *trans*-cinnamic acid monitored at 290 nm (Edwards and Kessmann, 1992). BA2H activity was determined by quantifying the SA synthesized from BA as described by Pan et al. (2006). The SA content in the reaction mixture was quantified to measure the free SA content. The SA synthesized from BA was calculated from the SA present in the assay mixture by subtracting the SA present in the crude supernatants. The protein concentration in the supernatants was determined by using a Bio-Rad protein assay kit (Bio-Rad Laboratories, Inc., Hercules, CA, USA), and BSA was used as a standard.

### Analysis of Chlorophyll Fluorescence, Gas Exchange, and Plant Dry Mass

Chlorophyll fluorescence was measured by using imaging PAM (MAXI; Heinz Walz, Effeltrich, Germany). The whole area of the third leaf from the bottom was used for the experiment. The seedlings were stored in the dark for at least 30 min before the measurements were taken. The intensities of the actinic light and saturating light settings were 280 and 4000 μmol m^-2^ s^-1^, respectively. The *F*v/*F*m, NPQ of chlorophyll fluorescence, and the *Φ*_PSII_ were measured and calculated in accordance with the method described by van Kooten and Snel (1990). *F*v/*F*m = (*F*m - *F*o)/*F*m; NPQ = (*F*m - *F’*m)/*F’*m; and *Φ*_PSII_ = (*F’*m - *F*s)/*F’*m. The light-saturated rate of CO_2_ assimilation (*A*_sat_) was measured with an open gas exchange system (LI-6400 XT; LI-COR, Lincoln, NE, USA) on the third leaf of each plant with a CO_2_ concentration of 380 μmol mol^-1^, a saturating PPFD of 1000 μmol m^-2^ s^-1^, a leaf temperature of 25 ± 1.5°C, and a relative air humidity of 80–90%. Plant dry mass was determined after drying the plant at 80°C to a constant weight.

### Determination of Electrolyte Leakage

To determine electrolyte leakage caused by chilling stress, the third fully expanded leaves were measured after chilling treatment in accordance with a previously described method with minor modifications (Hong et al., 2003). In brief, 0.1 g of leaf samples were cut into 1 cm^2^ fragments, rinsed with deionized water, and then shaken for 3 h at 22°C. The conductivity was then measured as EL1 by using an electrical conductivity meter (SG23; Mettler Toledo, Shanghai, China). The electrolyte leakage was calculated as a percentage of the total conductivity (EL2) measured after the leaf fragments were boiled for 15 min. Electrolyte leakage (%) = EL1/EL2 × 100.

### Measurements of Glutathione and Ascorbate Contents

Glutathione, oxidized glutathione (GSSG), AsA, and DHA contents were measured as previously described (Jiang et al., 2013).

### Antioxidant Assays

For antioxidant enzyme assays, leaf tissues (0.3 g) were ground with a 2 mL ice-cold buffer containing 50 mM PBS (pH 7.8), 0.2 mM EDTA, 2 mM AsA, and 2% (w/v) PVP. Homogenates were centrifuged at 12,000 ×*g* for 20 min, and the resulting supernatants were used to determine the enzyme activity. Peroxidase (POD) activity was measured as an increase in *A*_470_ by using guaiacol as a substrate (MacAdam et al., 1992). APX activity was measured as a decrease in *A*_290_ as described by Nakano and Asada (1981). Catalase (CAT) activity was measured as a decline in *A*_240_ in accordance with the method described by Patra et al. (1978). Total antioxidant capacity (T-AOC) was detected by measuring the ability to reduce Fe^3+^ to Fe^2+^ by using a total antioxidant capacity assay kit (Nanjing Jiancheng Bioengineering Institute, Nanjing, China) in accordance with the manufacturer’s instructions. All spectrophotometric analyses were conducted on an Infinite M200 PRO Multi-Detection Microplate Reader (Tecan, Männedorf, Zürich, Switzerland).

### Total RNA Extraction and Gene Expression Analysis

Total RNA was isolated from watermelon leaves by using TransZol reagent (TransGen Biotech, Inc., Beijing, China) in accordance with the manufacturer’s protocol. After extraction, the total RNA was dissolved in diethylpyrocarbonate-treated water. The cDNA template for quantitative real-time PCR (qRT-PCR) was synthesized from 1 μg of total RNA by using HiScript II Q RT SuperMix for qPCR (+g DNA wiper) (Vazyme, Piscataway, NJ, USA).

For qRT-PCR analysis, we amplified the PCR products in triplicate by using 1× Top Green qPCR SuperMix (TransGen Biotech, Inc., Beijing, China) in 10 μL qRT-PCR assays. PCR was performed using the LightCycler480 System (Roche, Basel, Switzerland), and the cycling conditions consisted of denaturation at 94°C for 30 s, followed by 40 cycles of denaturation at 95°C for 5 s, annealing at 55°C for 15 s, and extension at 72°C for 10 s. The multiple reference gene set of *ClEF1*α, *ClACT*, and *ClUBCP* was used as an internal control (Kong et al., 2014). Gene-specific primers for the *ClPAL* gene family were designed as previously described (Dong and Shang, 2013), and those for other genes were designed using the watermelon coding DNA sequences (CDSs) (v1) in the Cucurbit Genomics Database^1^. These primers were employed for amplification (Supplementary Table S1). The relative gene expression was determined as previously described by Livak and Schmittgen (2001).

### Statistical Analysis

The experiment involved a completely randomized block design with four replicates. Each replicate contained 10 plants. Statistical analysis of the bioassays was performed using the SAS statistical package. The differences between the treatment means were separated using Tukey’s test at a significance level of *P* < 0.05.

## Results

### SA Biosynthesis in Response to Chilling Stress in Watermelon

**FIGURE 1:**
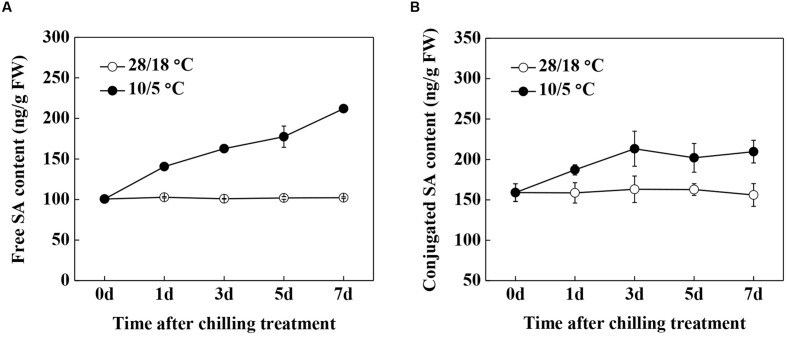
**Time course accumulation of SA in response to chilling stress in watermelon. (A)** Free SA content. **(B)** Conjugated SA content. Leaf samples were collected at the indicated times. The data are the means of four replicates with SEs. Different letters indicate significant differences between the treatments according to Tukey’s test (*P* < 0.05).

**FIGURE 2:**
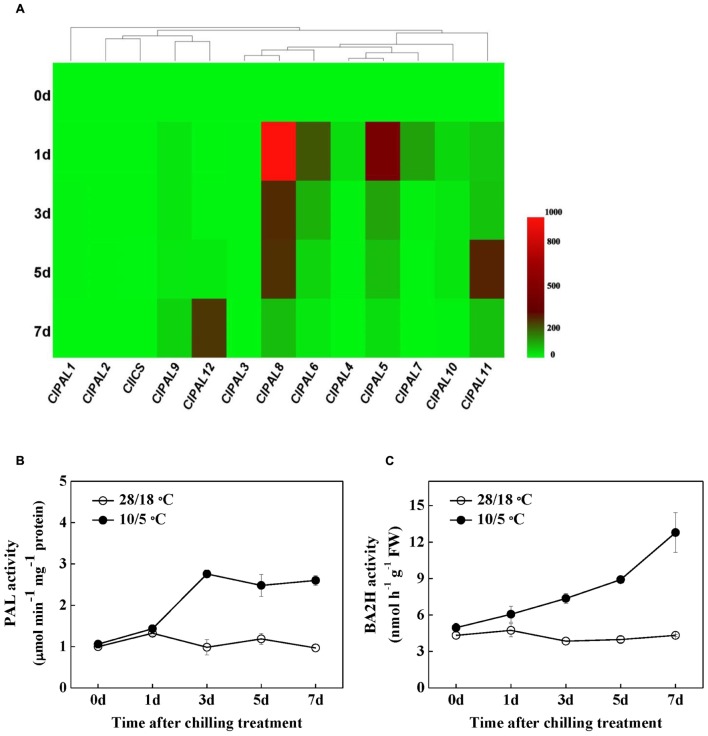
**The response of SA biosynthesis to chilling stress. (A)** Changes in the relative mRNA levels of *PAL* and *ICS* genes using matrix cluster analysis. The intensity of the red or green color represents the extent of up-regulation of the tested gene, and the dendrogram indicates the degree of similarity between the expression profiles of the tested genes. **(B,C)** Changes in the activities of PAL and BA2H enzymes in response to chilling stress in watermelon plants. Leaf samples were collected at the indicated times. The data are the means of four replicates with SEs. Different letters indicate significant differences between the treatments according to Tukey’s test (*P* < 0.05).

We first examined the free and conjugated SA contents during chilling stress to determine the involvement of SA in chilling stress response in watermelon (**Figures 1A,B**). Although, the levels of free and conjugated SA were constant throughout a 7-day period in plants and remained at normal conditions, the contents of both forms increased within 1 day after adjusting the temperature to 10/5°C and then remained high for 7 days. Gene expression analysis showed that the transcription levels of the C*lPAL* gene family were significantly induced after the onset of chilling treatment, with the exception of *ClPAL1*, *ClPAL2*, and *ClPAL3* (**Figure 2A**). As the transcripts of *ClPAL4*, *ClPAL5, ClPAL6*, *ClPAL7*, *ClPAL8*, and *ClPAL10* peaked at 1 day after chilling treatment, the remaining contents of *ClPAL9*, *ClPAL11*, and *ClPAL12* reached their highest transcription levels after 5 or 7 days. By contrast, the expression of *ClICS* slightly decreased upon chilling stress (**Figure 2A**). Exposure to chilling stress significantly increased the activities of PAL and BA2H, which are involved in the PAL pathway for SA biosynthesis (**Figures 2B,C**). These results suggest that SA is likely to be synthesized by the PAL pathway under chilling stress in watermelon plants.

### Effects of SA Levels on Chilling Tolerance in Watermelon

**FIGURE 3:**
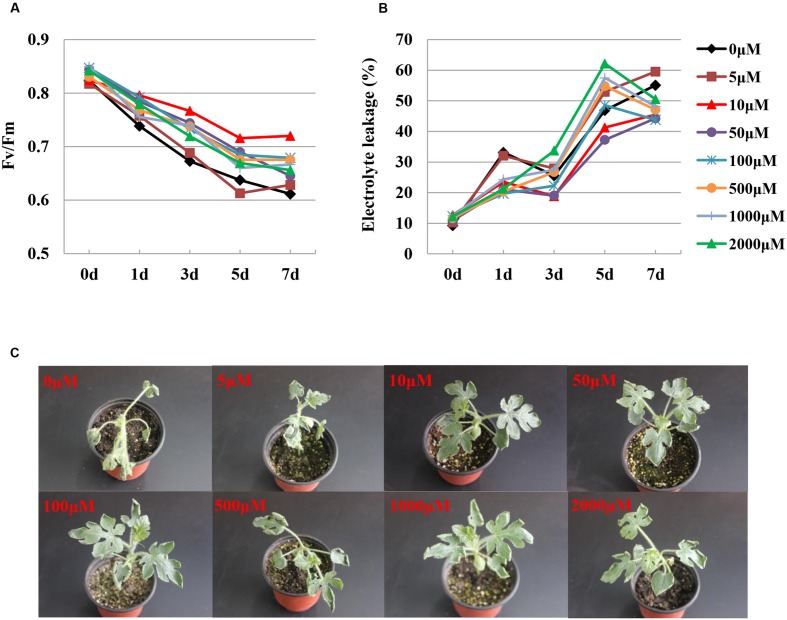
**Chilling tolerance phenotypes in different concentrations of SA-treated watermelon plants. (A)** Average *F*v/*F*m values in response to chilling stress. **(B)** Electrolyte leakage in response to chilling stress. **(C)** Phenotypes of SA-treated plants under chilling stress. *F*v/*F*m and electrolyte leakage were measured at the indicated times after chilling treatment. The data are the means of four replicates. The picture of representative plants was taken after 7 days of chilling treatment.

The *F*v/*F*m is a useful indicator of ROS-induced PS II damage in plants, whereas electrolyte leakage is commonly used to evaluate lipid peroxidation. To analyze the SA-induced changes in *F*v/*F*m and electrolyte leakage after chilling stress, different concentrations (0–2000 μM) of SA were pretreated for 24 h before the watermelon plants were presented at 10/5°C for 7 days. The *F*v/*F*m was higher in the SA-treated watermelon than in the water-treated plants under chilling stress. At 7 days after chilling treatment, the *F*v/*F*m of the water-treated plants decreased by 25.8%; however, the *F*v/*F*m of the SA-treated plants decreased by only 12.6–23.1%, with the concentration of 10 μM SA exerting the most significant effect (**Figure 3A**). Relatively low (≤5 μM) or high (≥500 μM) concentrations of SA increased the electrolyte leakage, whereas optimal concentrations (10–100 μM) of SA reduced the electrolyte leakage in watermelon leaves compared with the water-treated plants under chilling stress (**Figure 3B**). Symptoms of chilling-induced dehydration were detected on the leaves of the water-treated plants. Plants treated with low (≤5 μM) or high (≥500 μM) concentrations of SA showed wilting in the older leaves, whereas plants treated with moderate concentrations (10–100 μM) exhibited mild wilting after the chilling stress treatment (**Figure 3C**).

**FIGURE 4:**
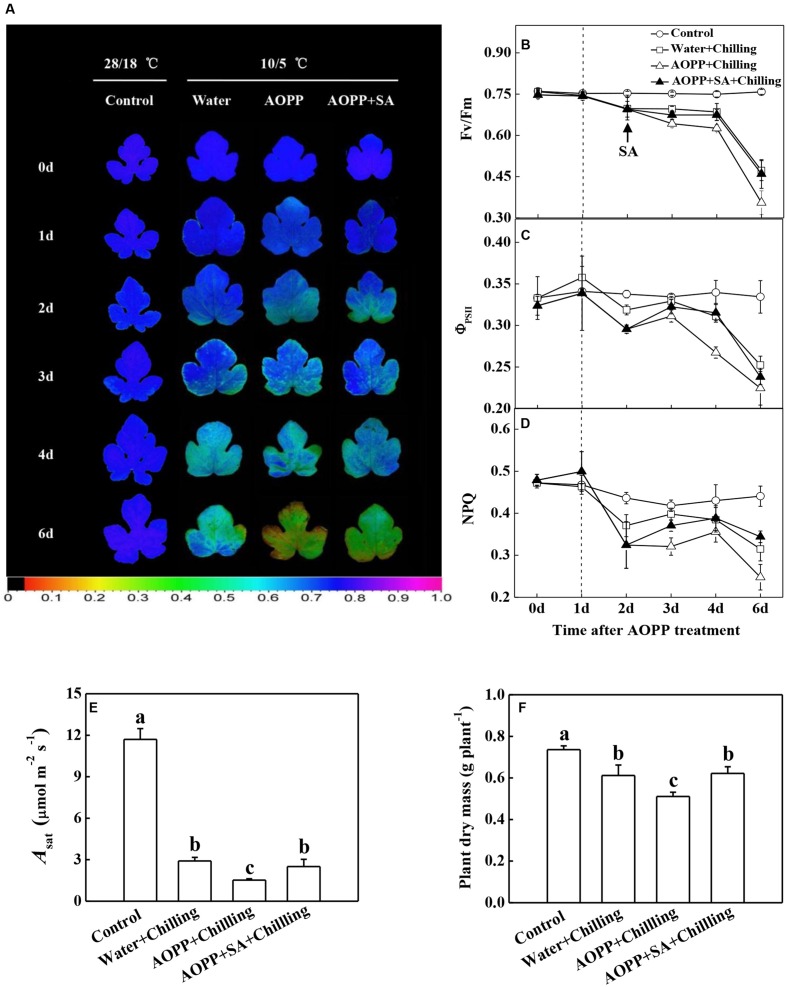
**Effects of chilling and SA on the photosynthetic capacity and growth in watermelon plants. (A)** Images of *F*v/*F*m. **(B)** *F*v/*F*m values. **(C)** *Φ*_PSII_ values. **(D)** NPQ values. **(E)** Light-saturated rate of CO_2_ assimilation (*A*_sat_). **(F)** Plant dry mass. The vertical dashed line in **(B–D)** indicates the transfer of the plants from 28/18°C to 10/5°C. Leaf samples were collected at the indicated times for chlorophyll fluorescence analysis. *A*_sat_ and plant dry mass were measured at 5 days after chilling treatment. The data are the means of four replicates with SEs. Different letters indicate significant differences between the treatments according to Tukey’s test (*P* < 0.05).

To examine the effects of SA on photosynthetic capacity and growth under chilling stress, the cellular SA levels were altered by applying a SA biosynthesis inhibitor (AOPP) and 10 μM exogenous SA. Chilling resulted in photoinhibition in the water-treated plants, as indicated by the 40.0% decrease in the *F*v/*F*m compared with the control after 5 days of treatment (**Figures 4A,B**). In addition, the *Φ*_PSII_ and NPQ in the water-treated plants decreased by 26.5 and 28.9% after 5 days of chilling stress, respectively (**Figures 4C,D**). Importantly, AOPP treatment induced significant decreases in the *F*v/*F*m, *Φ*_PSII_, and NPQ after 5 days of chilling stress, which decreased by 52.0, 33.8, and 44.4% compared with the control, respectively. However, the reduction of *F*v/*F*m, *Φ*_PSII_, and NPQ in the AOPP-treated plants could be recovered by SA application to levels similar to those in the water-treated plants under chilling stress (**Figures 4A–D**). Consistent with the changes in chlorophyll fluorescence parameters, the light-saturated rate of CO_2_ assimilation (*A*_sat_) and plant dry mass were reduced by 75.1 and 16.8% in the water-treated plants after 5 days of chilling treatment, respectively, in comparison with the control group. However, AOPP treatment of the chilling-stressed plants worsened the negative effects on photosynthesis and growth as indicated by the decrease in *A*_sat_ and plant dry mass to 87.0 and 30.6%, respectively, compared with the control group. Similarly, the application of 10 μM SA limited the decreases in the *A*_sat_ and plant dry mass to 78.6 and 15.5%, respectively, compared with the control group (**Figures 4E,F**). These results indicate that SA protects against chilling-induced oxidative damage.

### Chilling-Induced Changes in Redox Homeostasis Influenced by SA

**FIGURE 5:**
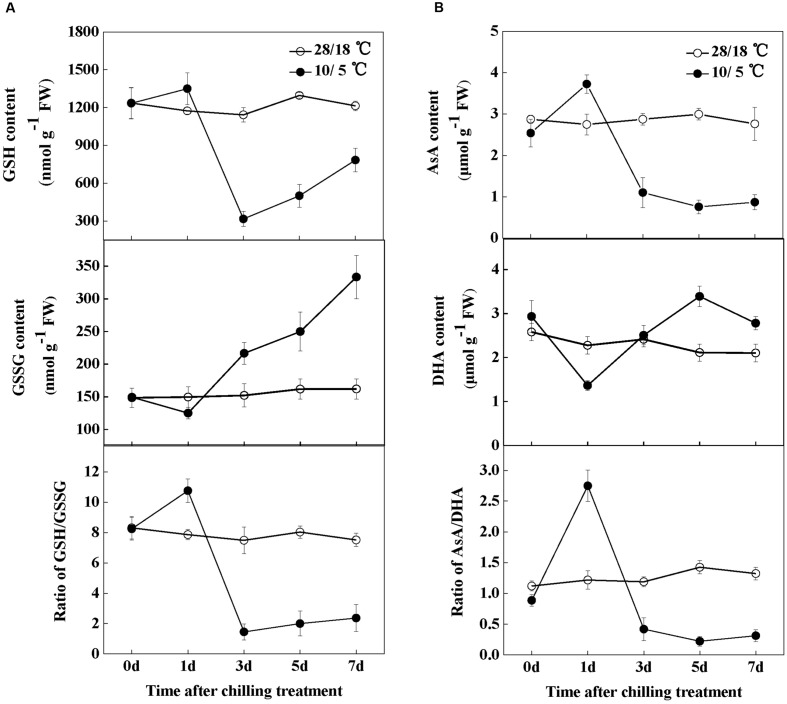
**Changes in glutathione and ascorbate homeostasis in response to chilling stress in watermelon plants. (A)** Changes in glutathione homeostasis. **(B)** Changes in ascorbate homeostasis. Leaf samples were collected at the indicated times. The data are the means of four replicates with SEs.

Both glutathione and ascorbate are multifunctional metabolites that play important roles in redox homeostasis and signaling. In this study, the effects of chilling stress on the contents of glutathione and ascorbate were analyzed. In general, the unchilled plants showed slight changes in GSH and GSSG contents. However, chilling increased the GSH content and decreased the GSSG content, which consequently significantly increased the GSH/GSSG ratio after 1 day (**Figure 5A**). Exposure to chilling stress considerably decreased the leaf GSH/GSSG ratio after 3 days of chilling treatment. This decrease was mostly attributable to a sharp reduction in GSH content and an increase in GSSG content (**Figure 5A**). Similar to the observed changes in glutathione content, the AsA and DHA contents showed no significant changes throughout the 7-day period in the absence of stress. By contrast, chilling significantly increased the AsA content but decreased the DHA content after 1 day of treatment. Subsequently, AsA considerably decreased with a concomitant increase in DHA content (**Figure 5B**). Thus, changes in the AsA/DHA ratio showed a pattern similar to that in the GSH/GSSG ratio (**Figures 5A,B**).

**FIGURE 6:**
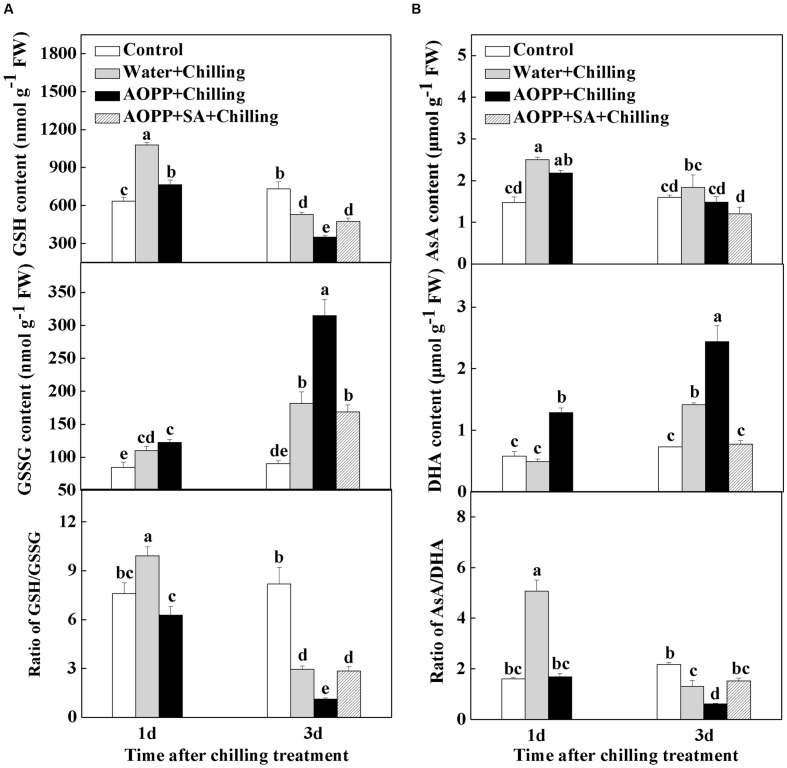
**The effects of chilling and SA on glutathione (A) and ascorbate (B) redox status in watermelon plants.** Water, plants sprayed with distilled water; AOPP, plants treated with 50 μM AOPP; AOPP + SA, plants pretreated with 50 μM AOPP for 1 day before chilling stress and then 10 μM SA after 1 day of chilling stress. Leaf samples were collected at the indicated times. The data are the means of four replicates with SEs. Different letters indicate significant differences between the treatments according to Tukey’s test (*P* < 0.05).

Previous studies suggested that the SA levels are closely related to cellular redox status (Chen et al., 1993; Vanacker et al., 2000; Noctor et al., 2002; Mou et al., 2003). To examine how SA regulates the cellular redox homeostasis, we determined the effects of AOPP and SA application on glutathione and ascorbate redox status upon chilling stress (**Figure 6**). At 1 day after chilling treatment, both the ratios of GSH/GSSG and AsA/DHA significantly increased by 30.6 and 214.3% in the water-treated than in the control plants. However, the GSH/GSSG and AsA/DHA ratios in the AOPP-treated plants were compromised to 82.9 and 105.0% of the control plants, respectively. Exposure to chilling decreased the GSH/GSSG and AsA/DHA ratios by 64.0 and 40.4% after 3 days, respectively, and this decrease was more significant in the AOPP-treated plants by 86.3 and 72.0%, respectively, in comparison with the control group. Moreover, the GSSG and DHA contents in the AOPP-treated plants significantly decreased after SA treatment. Thus, the GSH/GSSG and AsA/DHA ratios decreased by only 65.3 and 29.8%, respectively, compared with the control group. Overall, the results indicate that the cellular redox status is highly sensitive to chilling and that the SA levels strongly influence the cellular redox homeostasis in response to chilling stress.

### Responses of Antioxidant Metabolism to Chilling Stress

**FIGURE 7:**
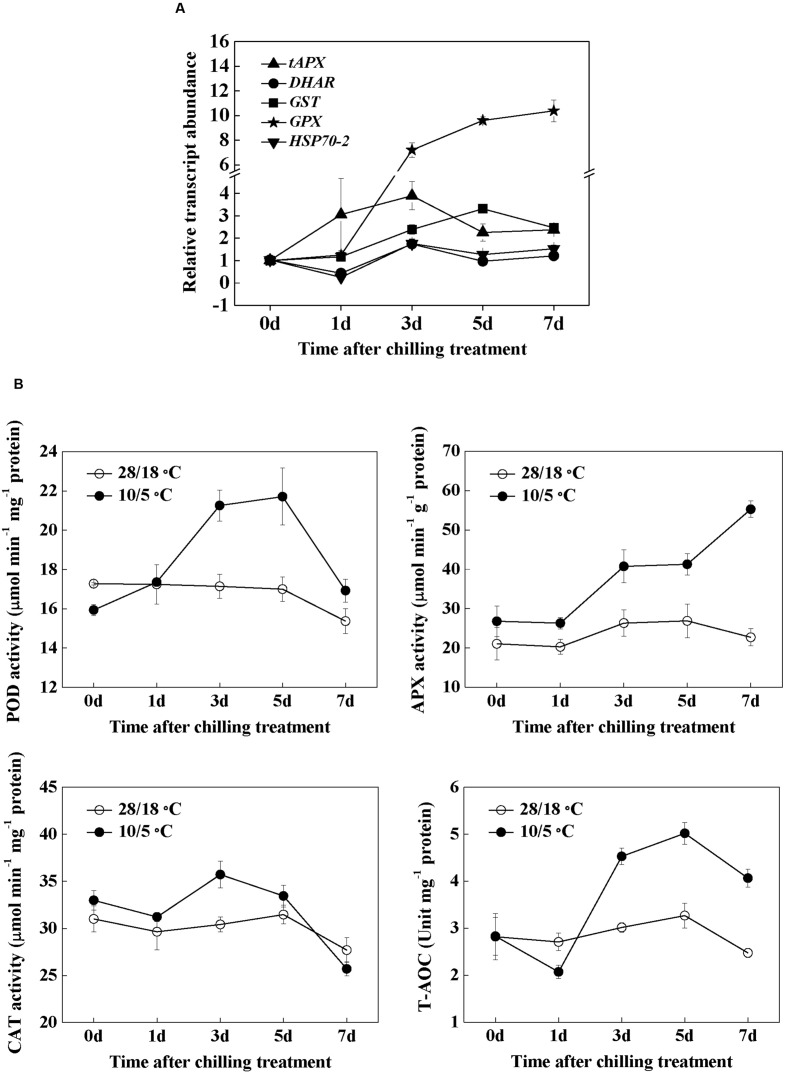
**The time-course response in gene expression of antioxidant genes (A) and activities of antioxidant enzymes (B) to chilling stress in watermelon.** Leaf samples were collected at the indicated times. The data are the means of four replicates with SEs.

To determine the antioxidative response to chilling stress in watermelon, we examined the changes in the transcript levels of five antioxidant genes (**Figure 7A**). Among the transcripts measured in these studies, *tAPX* (encoding thylakoid ascorbate peroxidase) increased early within 1 day, and the maximum level was observed at 3 days after chilling treatment. The enhanced transcript levels of *GST* (encoding glutathione-*S*-transferase) to chilling stress were observed after 3 days and peaked after 5 days; *GPX* (encoding glutathione peroxidase) transcripts significantly increased after 3 days and remained elevated up to 7 days after chilling treatment. Both the genes encoding dehydroascorbate reductase and heat shock protein 70-2 were upregulated 3 days after the temperature was adjusted to 10/5°C. Subsequently, these two transcripts declined to the levels of the controls without chilling treatment.

The activities of POD, CAT, and APX significantly increased 3 days after chilling treatment (**Figure 7B**). The activities of POD and CAT declined to basal levels after 7 days of chilling treatment, and the APX activity remained elevated in the next 4 days. Finally, the T-AOC significantly increased after 3 days, peaked after 5 days, and then declined after 7 days. The increase is accompanied by severe oxidative damage after chilling treatment (**Figures 3** and **7B**).

### Expression of *CBF* and *COR* Genes Influenced by Chilling and SA in Watermelon

**FIGURE 8:**
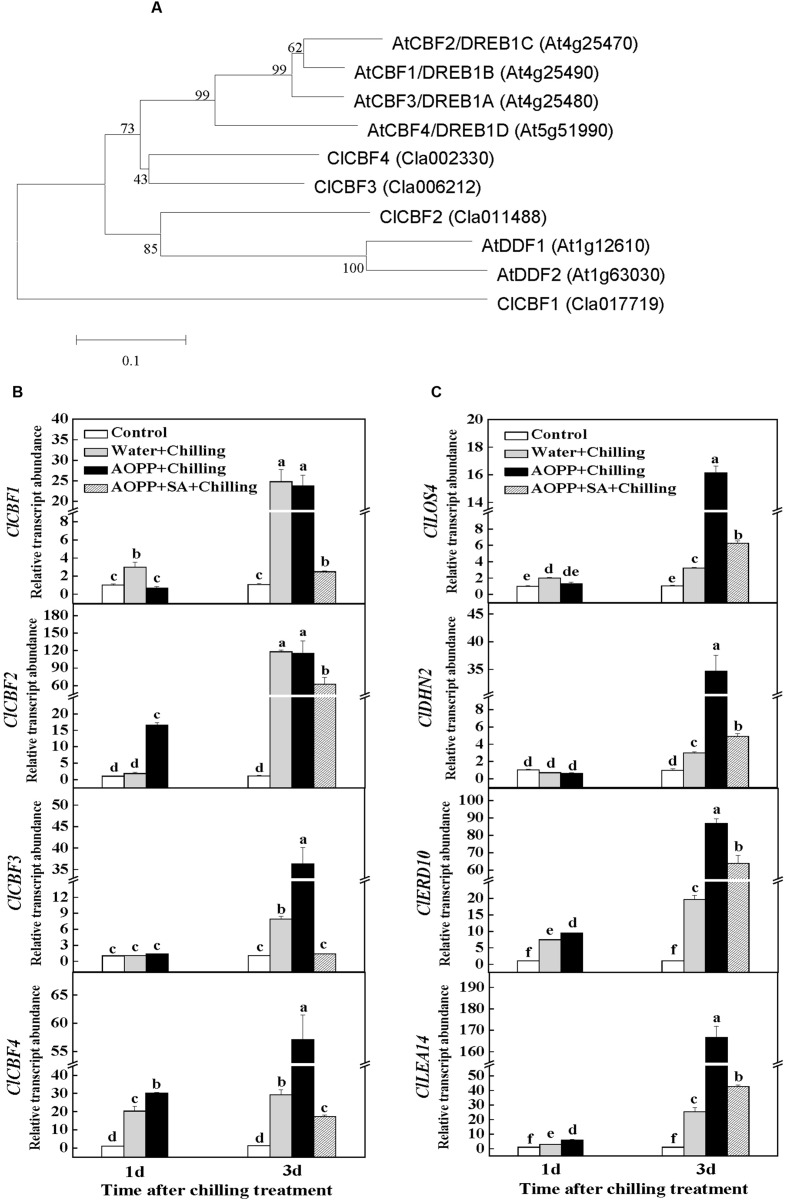
**The effects of chilling and SA on the expression of *CBF* and cold-responsive (*COR*) genes in watermelon plants. (A)** Phylogenetic tree of CBFs from *Citrullus lanatus* (Cl) and those identified DREB1/CBF proteins from *Arabidopsis*. The phylogenetic tree was constructed using MEGA 5 with the Neighbor–Joining method. Bootstrap values calculated from 1000 trials are shown at each node. The extent of divergence according to the scale (relative units) is indicated at the bottom. Predicted mature polypeptides lacking the putative transit peptide were employed for tree construction. **(B)** Changes in the expression of *CBF* genes in watermelon. **(C)** Changes in the expression of *COR* genes in watermelon. Leaf samples were collected at the indicated times. The data are the means of four replicates with SEs. Different letters indicate significant differences between the treatments according to Tukey’s test (*P* < 0.05).

To date, the most elucidated cold acclimation signaling pathway is the ICE1-CBF-COR transcriptional cascade, which is positively involved in cold stress tolerance among plants. A Cucurbit Genomics Database search based on sequence similarity with the predicted DREB1/CBF (A-1) subgroup of the AP2/ERF (APETALA2/DREB1) transcription factor family in *Arabidopsis* identified four CBF nucleotide sequences in *C. lanatus*: *ClCBF1* (Cla017719), *ClCBF2* (Cla011488), *ClCBF3* (Cla006212), and *ClCBF4* (Cla002330). A phylogenetic tree built from the alignment of these four proteins with the previously identified *Arabidopsis* DREB1/CBF proteins revealed the evolutionary distances between the sequences (**Figure 8A**).

To examine how the transcription of these *CBF* genes is influenced by chilling and SA content, their transcript levels were determined after chilling treatment in the water-, AOPP-, and SA-treated plants (**Figure 8B**). At 1 day after chilling treatment, the transcript levels of *ClCBF1* and *ClCBF4* in the water-treated plants increased by 3- to 20-fold, whereas those of *ClCBF2* and *ClCBF3* showed no differences compared with the control plants. However, the transcripts of all the tested *CBF* genes increased by 8- to 120-fold in the water-treated plants after 3 days of chilling treatment. Among the transcripts, the increase in *ClCBF3* and *ClCBF4* levels was more significantly induced by a combination of AOPP and chilling treatment. Interestingly, the induction of the *CBF* genes by the water- or AOPP-treated plants in combination with chilling treatment for 3 days was compromised in the AOPP+SA-treated plants because the transcript levels similar to the control (*ClCBF3*) or increased by only 2- to 60-fold (*ClCBF1*, *ClCBF2*, and *ClCBF4*). Moreover, AOPP application and chilling for 3 days induced a 15-fold increase in the transcript level of *LOS4* (low expression of osmotically responsive gene 4), encoding a nuclear localized RNA helicase, which may directly control the stability or other aspects of *CBF* transcripts under cold treatment (**Figure 8C**). Similarly, the increased levels of *LOS4* transcripts were compromised by the application of SA.

A DNA motif enrichment analysis indicated that the promoters of *ClDHN2* (encoding a dehydrin protein), *ClERD10* (encoding an early responsive dehydration protein), and *ClLEA14* (encoding a late embryogenesis abundant protein) were highly enriched in the CBF-binding site CCGAC (CRT/DRE regulatory element). This finding suggests that these genes are significant direct targets of the CBF transcription factors. Similar to the changes in the transcript levels of *ClCBF3* and *ClCBF4*, chilling increased the transcript levels of *ClDHN2*, *ClERD10*, and *ClLEA14* by 2- to 24-fold in the water-treated plants after 3 days. However, a combination of AOPP and chilling treatment resulted in a more significant increase in the transcript levels for all the tested *COR* genes. Importantly, this increase was again compromised in the subsequently SA-treated plants (**Figure 8C**).

## Discussion

### SA is Involved in the Regulation of Cold Responses in Watermelon

Multiple abiotic stress factors modulate major enzymes that are involved in SA biosynthesis in plants. Both water deficit and UV-B radiation cause SA accumulation as a result of increased activities of PAL and BA2H (Bandurska and Cieslak, 2013). Furthermore, Chen et al. (2008) reported that enhanced PAL activity is involved in heat pretreatment-induced chilling tolerance in *Musa* plants. Similarly, watermelon plants under chilling conditions persistently accumulated SA in free and conjugated forms (**Figure 1**). Interestingly, we found that chilling-induced SA production can be attributed to the PAL pathway because of the enhanced gene expression of *PAL4/5/6/7/8/10* and the enzymatic activities of PAL and BA2H; however, the mRNA level of ICS did not increase (**Figure 2**). By contrast, Kim et al. (2013) have recently reported that SA biosynthesis at low temperature in *Arabidopsis* proceeds through the ICS enzymatic pathway. In the present study, our data demonstrated that SA is required in an appropriate response to chilling stress, similar to plant responses to other environmental stresses (Ogawa et al., 2005; Bandurska and Cieslak, 2013; Krishnan and Merewitz, 2015).

Chilling stress-induced reduction in photosynthesis has been linked to changes in photosynthetic apparatus efficiency, photosynthetic enzymes, membrane properties, and non-enzymatic and enzymatic antioxidant system activities. In the present study, we demonstrate that watermelon plants are sensitive to chilling, as indicated by the photoinhibition-induced sharp decrease in the *F*v/*F*m (**Figure 3A**). Moreover, chilling-stressed watermelon maintained elevated levels of electrolyte leakage, which could be associated with increased membrane permeability (**Figure 3B**). Exogenous SA induces cold tolerance in a dose-dependent manner; low SA concentrations alleviate the chilling injury, whereas high concentrations and the continual application of SA cause severe growth damage and decrease cold tolerance capacity (Senaratna et al., 2000; Taşgín et al., 2003; Horváth et al., 2007). Our results indicated that moderate concentrations of SA (10–100 μM) promoted tolerance to chilling stress in watermelon, as indicated by the reduced electrolyte leakage and increased *F*v/*F*m; leaves from plants sprayed with low (≤5 μM) or high (≥500 μM) concentrations of SA did not show any alteration in their chilling tolerance (**Figure 3**). PS II is prone to photooxidative damage when exposed to chilling or high light intensity. Recent evidence has also suggested that SA is an important regulator of photosynthesis and PS II under abiotic stresses (Mohammed and Tarpley, 2013; Li et al., 2014; Zhang et al., 2015). Similar to the results observed under high light stress (Mateo et al., 2006), the photoinhibition and photodamage under chilling stress were more severe by the inhibition of SA production with AOPP (**Figures 4A–D**). Meanwhile, the AOPP-induced decrease in the photosynthetic capacity and growth under chilling stress was limited by SA application (**Figures 4E,F**). Thus, SA-improved photosynthesis and growth in watermelon are largely attributed to the reduction of photooxidation that occurred around PS II.

### Cellular Redox Signaling is Correlated with SA-Induced Chilling Tolerance in Watermelon

Networks of redox signaling in cells play essential roles in the acclimation of plants to abiotic stresses. As redox active compounds, AsA and GSH maintain cellular homeostasis by regulating important biological pathways, such as gene expression, energy metabolism, and cell division under stress conditions (Foyer and Noctor, 2011). In the current study, chilling increased the GSH and AsA contents but decreased the GSSG and DHA contents after 1 day, which substantially increased the GSH/GSSG and AsA/DHA ratios (**Figure 5**). Interestingly, the time-course responses of the GSH/GSSG and AsA/DHA ratios to chilling preceded the expression of antioxidant genes and the activities of antioxidant enzymes (**Figures 5** and **Figure 7**). This result supports that the changes in GSH/GSSG and AsA/DHA are involved in the activation of antioxidant defense mechanisms through a redox signaling chain in response to chilling stress (Kocsy et al., 2001). Both endogenous and exogenous SA plays important roles in antioxidant metabolism and demonstrates a tight control over cellular ROS (Kang et al., 2014; Khan et al., 2014). In wheat, SA signaling correlates with AsA- and GSH-related mechanisms to improve Cd and salinity tolerance (Li et al., 2013; Kovács et al., 2014). In the present research, the increased GSH/GSSG and AsA/DHA ratios in chilling-treated plants can be effectively abolished by AOPP, and the changes in the GSH/GSSG and AsA/DHA ratios were closely related to the alteration in SA content (**Figure 6**). These results suggest that cellular redox signaling is strongly implicated in SA-induced chilling tolerance.

### SA Signaling Might Negatively Regulate the CBF-Dependent Cold-Responsive Pathway during Chilling Stress in Watermelon

In recent years, models of phytohormone signaling involved in the regulation of plant growth and abiotic stress responses have been established by genetic and biochemical approaches, and growing evidence has indicated that hormonal components play important roles in regulating plant cold tolerance by either CBF-dependent or CBF-independent pathways (Shi et al., 2015). Exposure to chilling stress after 3 days increased the transcript abundance of all the tested *CBF* genes in the water-treated plants (**Figure 8B**), suggesting the function similarity of CBFs in watermelon and *Arabidopsis* in response to chilling stress (Gilmour et al., 2004; Thomashow, 2010). Intriguingly, AOPP treatment with chilling enhanced the expression of *ClCBF3* and *ClCBF4* with concomitant induction of three *COR* genes (*ClDHN2*, *ClERD10*, and *ClLEA14*; **Figures 8B,C**). However, this stimulatory action was compromised after exogenous application of SA (**Figures 8B,C**). Similarly, the RNA helicase gene *LOS4*, which is important for nuclear mRNA (including *CBF*s) export in response to temperature stress (Gong et al., 2005), showed an enhanced expression with the pretreatment of AOPP compared with water-treated plants under chilling stress. Again, the AOPP-induced expression of *LOS4* was compromised after SA treatment (**Figure 8C**). A calmodulin-binding transcription activator, CAMTA3/AtSR1, recognizes the promoter of *CBF2/DREB1C* to positively regulate cold tolerance and the promoter of *EDS1* to repress disease resistance, suggesting that cold signaling and SA signaling are interrelated (Doherty et al., 2009; Du et al., 2009). In addition, a study on the SA-accumulating lines *siz1* and *acd6* in *Arabidopsis*, which exhibit a dwarf phenotype, confirmed that the sensitivity to cold stress is associated with increased endogenous SA accumulation and decreased expression of *DREB1A/CBF3* and its regulon genes (Miura and Ohta, 2010). These results suggest that SA signaling plays a negative role in the regulation of the CBF-responsive pathway under chilling stress.

**FIGURE 9:**
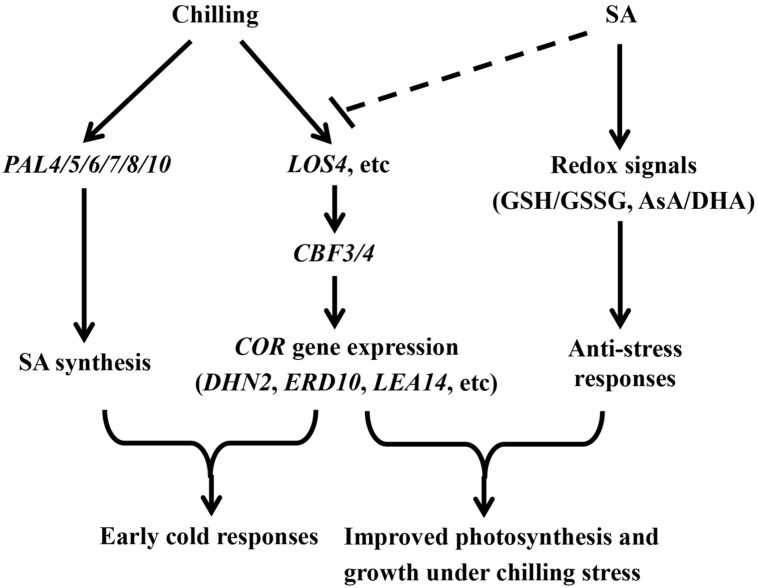
**A proposed model for SA-induced chilling tolerance in coordination with CBF-responsive pathway in watermelon plants**.

In summary, evidence is provided to support the concept that SA-induced chilling tolerance involves glutathione- and ascorbate-mediated redox signaling, which may positively regulate the expression of antioxidant genes and the activities of antioxidant enzymes in stress responses. Moreover, the coordination of SA signaling with CBF signaling was suggested to provide an appropriate defense response under chilling stress in watermelon (**Figure 9**). This study provides a new strategy to develop ideotypes for watermelon and improve the overall plant performance under low-temperature climate. However, further study is required to provide genetic evidence of the involvement of redox signaling in SA-induced chilling tolerance. Other mechanisms underlying SA-mediated chilling tolerance should also be explored to understand the relationship between SA and cold signaling.

## Author Contributions

FC conceived and designed the research; FC, JL, MG, QK, and YH performed the experiments and analyzed the data; KS provided bacterial cultures and supervised the study; FC and ZB wrote the manuscript. All authors read and approved the manuscript.

## Conflict of Interest Statement

The authors declare that the research was conducted in the absence of any commercial or financial relationships that could be construed as a potential conflict of interest.

## Footnotes

1 http://www.icugi.org

## Abbreviations

AOPP: L-a-aminooxy-b-phenylpropionic acid
AsA: reduced ascorbate
BA: benzoic acid
BA2H: BA 2-hydroxylase
CBF: C-repeat binding factor
COR: cold-responsive
DHA: dehydroascorbate
DREB1: dehydration-responsive element binding factor 1
Fv/Fm: maximum quantum yield of PS II
ICS: isochorismate synthase
NPQ: non-photochemical quenching
PAL: phenylalanine ammonia-lyase
ROS: reactive oxygen species
SA: salicylic acid
ΦPSII: effective quantum yield of PS II.
