# Supplementary material for: Redox Signaling and CBF-Responsive Pathway Are Involved in Salicylic Acid-Improved Photosynthesis and Growth under Chilling Stress in Watermelon
Source: Front Plant Sci. 2016 Oct 10;7:1519. doi: 10.3389/fpls.2016.01519 (PMC5056192; doi:10.3389/fpls.2016.01519)
Supplement: Supplementary file 1 [file Table_1.DOC]

***Supplementary Material***

**Redox Signaling Cooperates with the CBF-Dependent Responsive Pathway in Salicylic Acid-Induced Chilling Tolerance in Watermelon**

**Running title:** Redox control of SA signaling

Fei Cheng^1^, Junyang Lu^1^, Min Gao^1^, Kai Shi^2^, Qiusheng Kong^1^, Yuan Huang^1^, Zhilong Bie^1^,^*^

^1^Key Laboratory of Horticultural Plant Biology, Ministry of Education/College of Horticulture and Forestry Sciences, Huazhong Agricultural University, Wuhan, China

^2^Department of Horticulture, Zhejiang University, Hangzhou, China

^*^**Correspondence:**

Zhilong Bie

E-mail: biezl@mail.hzau.edu.cn

**Table S1. Gene-specific primers designed for qRT-PCR.**

| Gene  name | Gene | Forward primer 5’-3’ | Reverse primer 5’-3’ |
| --- | --- | --- | --- |
| *ClPAL1* | Cla008727 | ATGAAACCCAACACTTGCAAT | ACTCCCCCACCATTTTCTT |
| *ClPAL2* | Cla011180 | AAGGCTGCCAACGCTCTCAG | ATCGCTTCGACGAGCAACGG |
| *ClPAL3* | Cla018297 | GCCGGAACAATATCAACCATG | CATCCGCTTCACTTCTTCC |
| *ClPAL4* | Cla018298 | TGCGCCATTACTACTCATCC | GCGCTTCACCTCATTGAG |
| *ClPAL5* | Cla018299 | TGACTTGAGGCACCTAGAAG | ATCTTGAAGGATGAAGTGTGC |
| *ClPAL6* | Cla018300 | TGGCACCAACTCAATTCCAG | CCTCATCGAGATGGCTTCC |
| *ClPAL7* | Cla018301 | TGCCCAATTGGAACAATGTG | TATACTCTTCCACCATCCTCTT |
| *ClPAL8* | Cla018302 | TGGAGACCATCTGCAAGAG | GCGCTTCACCTCATCAAG |
| *ClPAL9* | Cla018303 | CCCTAGTCAAGACCATTTGCAA | GCTTGACAAGAGGCCTCCGA |
| *ClPAL10* | Cla012779 | ATGGCCCAAAAGGTTTGTGCT | ATACTCTTCCACCATCCGC |
| *ClPAL11* | Cla012780 | GGCTTCCAAAAATAATGATTC | TCCTCCAAGCTTCACCAAA |
| *ClPAL12* | Cla013761 | ATCCGGAAAATGGCAACCAT | CCTCCAAGTCGCACAATAG |
| *ClICS* | Cla019128 | ACGGAGAGTCTGAGGAGAG | GCACTTGAAGCCGAATAATC |
| *CltAPX* | Cla013927 | TCCCTTCAGGAGGCATTATC | CACCAAGGTTGCTGAGTTTG |
| *ClDHAR* | Cla013224 | AATTGCTCGCTTTCTCGTTT | GTAATGGAAGCTTTGACGCA |
| *ClGST* | Cla007826 | ATCCTCTGTTGCCTCGTGAT | CCAACGAAGTTTCTCTTCTGG |
| *ClGPX* | Cla011457 | TTGAGATTCTTGCATTTCCG | GCATTGTTTCCATTCACGTC |
| *ClHSP70-2* | Cla000858 | GAAGGAGCTTGGAGACAAGG | CAGCTGCATCACCTCTTGAT |
| *ClCBF1* | Cla017719 | ACGAGTTATGTTGTCATCGTT | CTCCTCCGACATCAAAGAA |
| *ClCBF2* | Cla011488 | AAGTGATTCTGGCGTCCAACCG | ATTCCTCCGGCGTACTCCTCTG |
| *ClCBF3* | Cla006212 | GAAGACTACTTCTCCAGTTCCG | CTGGCCGCCAACATAATATCC |
| *ClCBF4* | Cla002330 | TTCCTCCCCACTTTGCCAC | TTGCCGGAGTTCCGCTGTCGC |
| *ClLOS4* | Cla017751 | CAGACTCTACCACCAGCAAC | CATCATCATCTTCTTCATCGC |
| *ClDHN2* | Cla014571 | GGCAATGTCATCTCCGAGAC | CGTCAACACCACCTTATCATG |
| *ClERD10* | Cla021949 | GCGGAGTACGAGAACAAG | ACGCCACTCTCTTGAACTC |
| *ClLEA14* | Cla021202 | AACATGCCGAAGCCAGAG | TGGAGACAGAGACATTGGC |
| *ClACT* | Cla007792 | CCATGTATGTTGCCATCCAG | GGATAGCATGGGGTAGAGCA |
| *ClEF1α* | Cla010539 | AGCACGCTCTTCTTGCTTTC | ACGATTTCGTCGTACCTTGC |
| *ClUBCP* | Cla010163 | ACCAACAGTCCGCTTTGTGT | ATTGGGCTCCACTGATTTTG |
